# A Case of Leukoencephalopathy Secondary to Methotrexate Toxicity

**DOI:** 10.7759/cureus.91458

**Published:** 2025-09-02

**Authors:** Ayema Haque, Channing Pezet, Aidrian Ranjith, Neil Hughes

**Affiliations:** 1 Internal Medicine, Western Michigan University Homer Stryker M.D. School of Medicine, Kalamazoo, USA

**Keywords:** leukoencephalopathy, methotrexate neurotoxicity, methotrexate toxicity, mucositis, psoriatic arthritis

## Abstract

Methotrexate (MTX) has known toxicities affecting the kidneys, mucous membranes, liver, lungs, and nervous system. Rarely does a patient take large amounts of MTX, as attempts are made to avoid these toxicities. This case presents a patient who ingested seven weeks' worth of MTX in one week and developed neurotoxicity, with MRI showing subacute infarcts in the right frontal lobe, right parietal, and occipital lobes in the right middle cerebral artery watershed distribution. Prompt leucovorin therapy improved symptoms of altered mental status. This case highlights the importance of accurate prescription instructions and details the neurotoxicity that can occur with high MTX ingestion.

## Introduction

Methotrexate (MTX) is a dihydrofolate reductase inhibitor commonly associated with adverse reactions, including hepatotoxicity, hematologic toxicity, stomatitis, pulmonary toxicity, neurologic toxicity, dermatological toxicity, nausea, and vomiting [1]. High-dose MTX treatment is usually reserved for the treatment of leukemia and lymphomas, osteosarcoma, and primary central nervous system (CNS) lymphoma [2]. These treatments are typically followed by leucovorin rescue right away to mitigate adverse effects. Rheumatologic diseases like psoriatic arthritis and rheumatoid arthritis use low-dose MTX therapy. MTX is commonly administered orally or as an injection and dosed weekly [1]. Rarely, patients will consume larger amounts of MTX and develop severe adverse effects. This case presents a patient who ingested the equivalent of seven weeks' worth of MTX in one week due to incorrect labeling on his medication. The patient developed leukoencephalopathy, mucositis, pulmonary fibrotic changes, rash, and neutropenia. This case describes the patient’s hospital course and treatment and their ultimately good outcome.

## Case presentation

**Figure 1:**
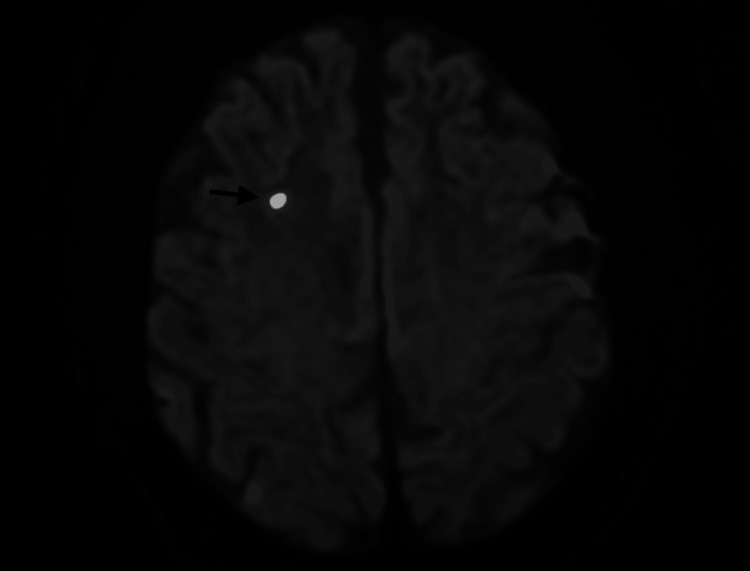
Foci of diffusion restriction are demonstrated in the subcortical white matter of the right frontal lobe (indicated by an arrow)

**Figure 2:**
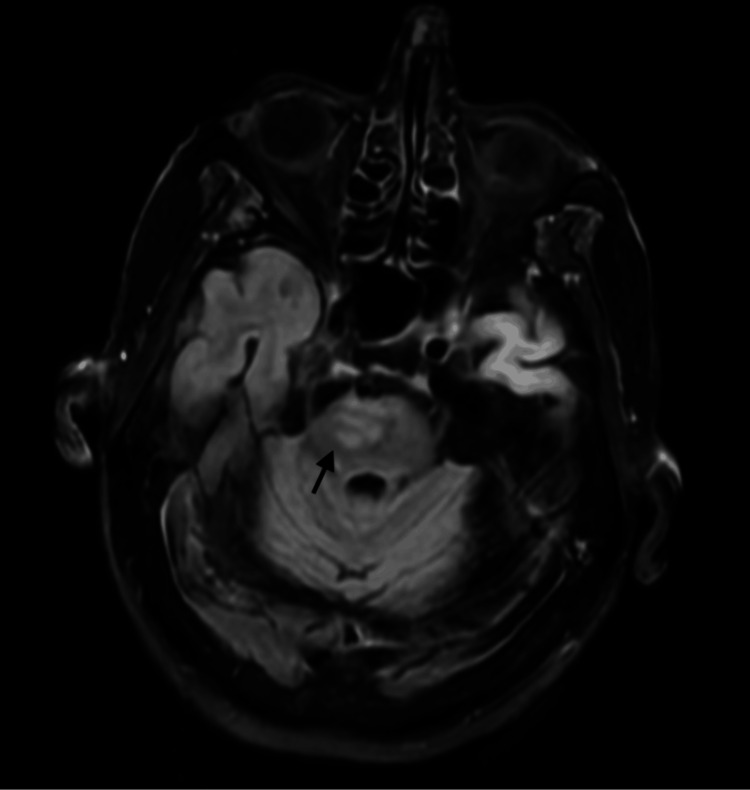
Interval development of edema in the ventral right pons (indicated by an arrow)

A 74-year-old male with a history of inflammatory arthritis (on hydroxychloroquine and MTX), degenerative disc disease, monoclonal B cell lymphocytosis, and chronic kidney disease presented with dysphagia, drooling, and dysarthria for the previous two days. On presentation, he was afebrile and tachycardic. He had oral mucositis and a diffuse pustular rash. Labs revealed leukopenia with an absolute neutrophil count of 0.1 thousand per microliter and an elevated creatinine (Table 1). CT of the head/neck showed increased enhancement associated with the mucosal surfaces of the hypopharynx, with concern for acute pharyngitis. Hematology/oncology consultation recommended against filgrastim. Herpes simplex virus (HSV)-1 test was positive, and acyclovir was promptly started. A chest CT revealed fibrotic changes and bullous emphysema. MRI of the head showed a subacute infarct in the right frontal lobe and right parietal and occipital lobes in the right middle cerebral artery watershed distribution or embolic disease, no hemorrhagic transformation, mild chronic microvascular ischemic disease, and edema of the right ventral pons (Figures 1, 2). A CT head and neck angiogram revealed areas of right vertebral artery stenosis without occlusion.

**Table 1:** Lab results on first presentation CO₂: carbon dioxide; AST: aspartate transaminase; ALT: alanine transaminase; LDH: lactate dehydrogenase; WBC: white blood cell count; RBC: red blood cell count

Lab	Result on initial presentation	Normal value range
Creatinine (mg/dL)	1.84	0.7 - 1.3
CO_2_ (mmol/L)	19	23 - 32
AST (U/L)	6	0 - 37
ALT (U/L)	11	6 - 45
Albumin (g/dL)	3.2	3.5 - 5
LDH (U/L)	894	94 - 250
Lactic acid (mmol/L)	2.5	0.7 - 2.5
WBC (10^9/L)	3.0	4 - 11
RBC (10^12/L)	1.57	4.4 - 6
Platelets (10^9/L)	25	140 - 440
Neutrophils (10^9/L)	0.1	1.1 - 6.1
Absolute reticulocyte count (10^9/L)	0.6	23 - 93

Further history from the daughter revealed MTX toxicity. The pharmacist reported that the patient was prescribed MTX 20 mg weekly, but it was transcribed to 20 mg daily. The patient's daughter reported that she saw the patient take seven weeks’ worth of MTX in one week. Poison control recommended starting leucovorin. Radiology confirmed MRI findings of the toxicity. Leucovorin was given for four days, and the symptoms drastically improved. Lumbar puncture was attempted twice; however, it was unsuccessful. Blood cultures on day 18 of hospitalization were positive for *Candida lusitaniae.* Intravenous micafungin was started for two weeks. Esophagogastroduodenoscopy done by gastroenterology showed no evidence of esophagitis. Transesophageal echocardiography showed no signs of vegetations. The patient was discharged to rehab with 400 mg daily of fluconazole. The patient followed up with his rheumatologist a month later, and his MTX and hydroxychloroquine were discontinued with a plan to treat his arthritis with nonsteroidal anti-inflammatory drugs. As the lumbar puncture was unsuccessful, it is uncertain if the encephalopathy was secondary to MTX toxicity or HSV encephalitis. However, the acuity of symptoms, MRI results, and strength of toxicity are suggestive of MTX toxicity.

## Discussion

MTX is a dihydrofolate reductase (DHFR) inhibitor commonly used for rheumatological conditions, including psoriatic arthritis, and malignancies such as acute lymphoblastic leukemia (ALL) and osteosarcoma. MTX’s role in inhibiting DNA synthesis leads to rapidly dividing cells being affected first. MTX toxicity, whether by accidental overingestion or side effect of therapy, can lead to mucocutaneous erosion and ulceration, interstitial pneumonitis, pancytopenia, leukoencephalopathy, hepatotoxicity, renal damage, and immunosuppression [1].

MTX toxicity can be reversed with leucovorin, a folinic acid analog that bypasses DHFR and allows DNA synthesis to continue, thereby decreasing MTX-related effects. MTX use in autoimmune diseases is explained by an alternate mechanism: it inhibits AICAR transformylase, decreasing purine metabolism and increasing adenosine amounts. Adenosine has anti-inflammatory action by decreasing T cell activation, downregulating B cells, and increasing activated CD-95 T cell sensitivity [1]. This mechanism can also lead to immunosuppression and opportunistic infection (*Pneumocystis jirovecii*, etc.) or reactivation of pathogens (HSV, varicella zoster virus, etc.).

When suspecting MTX toxicity, MTX serum levels should be measured and leucovorin rescue should be initiated promptly, ideally within 24 hours of the last MTX dose. MTX serum levels should be monitored to help identify the dosage and duration of leucovorin therapy required [3]. Patients should also avoid nephrotoxic medications and maintain adequate hydration to promote the renal excretion of MTX. Alkalinization of the urine with sodium bicarbonate increases the solubility of MTX and metabolites by five to eightfold, reducing the chance of crystal precipitation [4]. Patients may benefit from glucarpidase, a drug that hydrolyzes MTX into two inactive metabolites and can further decrease MTX concentrations [5].

Neurotoxicity is a more common occurrence with intrathecal administration and high-dose MTX; most literature describes cases of neurotoxicity in patients treated for ALL. The exact pathogenesis of acute MTX-related encephalopathy is unknown but thought to be related to increasing amounts of adenosine, causing vascular dilation and cerebral edema. Some patients had immediate reversal of neurotoxicity with aminophylline, an adenosine receptor antagonist [2]. Steroids may help decrease cerebral edema, and early leucovorin remains essential in the treatment of all MTX toxicities. There may also be a role for dextromethorphan, an N-methyl-D-aspartate (NMDA) receptor antagonist, in treating neurological dysfunction with MTX toxicity [6].

Mistakenly taking MTX daily instead of weekly was found to be the most common (54.8%) error associated with adverse consequences of low-dose MTX in a study conducted in France [7].

## Conclusions

MTX is a drug that has benefits in cancer and rheumatologic disease, but its toxicity can have a varied presentation. Early leucovorin treatment is the standard of care for MTX toxicity as it can bypass the inhibited DHFR enzyme. This case documented acute MTX encephalopathy as evidenced by MRI; however, a positive HSV-1 does not exclude HSV encephalitis as the cause of this patient’s neurologic disease. Prompt treatment of MTX-toxicity-related encephalopathy appears to be reversible short term; however, more research needs to be conducted to understand long-term complications of high-dose MTX toxicity effects on the brain.
